# Stakeholder Perspectives on Navigating Evidentiary and Decision Uncertainty in Precision Oncology

**DOI:** 10.3390/jpm12010022

**Published:** 2022-01-01

**Authors:** Samantha Pollard, Jessica Dunne, Sarah Costa, Dean A. Regier

**Affiliations:** 1 Cancer Control Research, BC Cancer, Vancouver, BC V5Z 4C2, Canada; spollard@bccrc.ca (S.P.); jessicadunne17@outlook.com (J.D.); sarahecosta@gmail.com (S.C.); 2 School of Population and Public Health, University of British Columbia, Vancouver, BC V6T 1Z3, Canada

**Keywords:** health technology assessment, precision oncology, qualitative interviews

## Abstract

(1) Background: Precision oncology has the potential to improve patient health and wellbeing through targeted prevention and treatment. Owing to uncertain clinical and economic outcomes, reimbursement has been limited. The objective of this pan-Canadian qualitative study was to investigate barriers to precision oncology implementation from the perspectives of health system stakeholders. (2) Methods: We conducted 32 semi-structured interviews with health technology decision makers (*n* = 14) and clinicians (*n* = 18) experienced with precision oncology. Participants were recruited using a purposive sampling technique. Interviews were analyzed using thematic analysis. Recruitment continued until two qualitative analysts reached agreement that thematic saturation was reached. (3) Results: While cautiously optimistic about the potential for enhanced therapeutic alignment, participants identified multiple decisional challenges under conditions of evidentiary uncertainty. Decision makers voiced concern over resource requirements alongside small benefitting patient populations and limited evidence supporting patient and health system impacts. Clinicians were comparatively tolerant of evidentiary uncertainty guiding clinical decision-making practices. Clinicians applied a broader definition of patient benefit, focusing on the ability to assist patients making informed clinical decisions. (4) Conclusions: Sustainable precision oncology must balance demand with evidence demonstrating benefit. We show that clinicians and decision makers vary in their tolerance for evolving knowledge, suggesting a need to establish evidentiary standards supporting precision oncology reimbursement decisions.

## 1. Introduction

Precision oncology, the application of comprehensive genomic profiling (CGP) to match patient pathophysiology to treatment, is producing a new generation of cancer therapeutics [1,2]. Through the application of CGP, precision oncology aims to improve patient health while increasing health system efficiency by deploying costly treatments to patients most likely to incur benefits through improved quality and length of life [2]. Clinical implementation efforts endeavour to broaden the scope of patient uptake and benefits, enabling patients and their healthcare providers to integrate CGP findings into complex care decision making [3].

With a demonstrated ability to identify targetable genomic aberrations, patient, public, and clinician appetite for broad system-level uptake of CGP is increasing [4,5,6,7]. Support for the integration of precision medicine into clinical care continues to receive public, governmental, and institutional support across publicly and privately funded health systems [5,6,7]. Despite this, precision oncology using CGP has largely been confined to research, due in part to limited evidence informing downstream patient and resource impacts [2,8,9,10,11].

Efforts to respond to widespread support for precision oncology through expanded reimbursement and clinical implementation are hampered by a lack of evidence. Good evidence requires high-quality data. Recent initiatives have led to an evolution of knowledge wherein cancers are characterized by their genomic features. Alongside an enhanced ability to, for example, estimate prognosis and anticipate treatment response, increased knowledge has opened the door to the development of targeted therapeutics for specific, sometimes small, patient subpopulations. The rarity of individual tumour aberrations presents challenges generating experimental evidence to inform causal inference. Traditional trial designs require large sample sizes, often considered infeasible within precision oncology given the lengthy accrual periods required for adequately powered analyses. As a result, large-scale evaluations involving randomization, prospective follow up, and the estimation of causal effects that apply traditional methods are uncommon [10].

A further challenge contributing to a limited evidence base reflects a highly variable patient care and resource use trajectory following CGP. Genomics-informed treatment uptake is a function of the presence or absence of clinically actionable findings, as well as the extent to which targeted treatments are available and accessible to patients. Individual patient trajectories following sequencing are further impacted by decisional factors that include but are not limited to patient values and treatment goals, and willingness to trade off risks for expected but often uncertain benefits. As a result, estimating the impact of CGP on patient outcomes and resource utilization is inherently complex. 

Comprehensive evaluations of precision oncology are challenging in light of limited patient numbers (e.g., rare genomic aberrations) and highly variable outcome care patterns following CGP. For these reasons, current evidence estimating value for money is sparse, resulting in evidentiary and decision uncertainty [12,13]. An evidence-base unable to provide robust impact estimates presents substantial barriers for jurisdictions accountable to public payers when allocating healthcare resources. Within Canada, the responsibility for healthcare provision is under the mandate of provincial and territorial governments, and thus reflects jurisdiction-specific priorities and budgetary constraints [14]. Reimbursement and clinical uptake are predicated on the availability of evidence enabling decision makers, healthcare providers, and patients to weigh individual and system-level risks against expected benefit [12,15,16,17]. Risks, within publicly funded systems, are not confined to patient level outcomes, but refer also to opportunity costs associated with allocating scarce resources toward treatments and interventions with uncertain value for money. Due to a paucity of evidence informing economic impact, clinical and resource allocation decisions within this context are fraught with uncertainty [15].

Currently, there exists a tension between broad support for precision oncology reflected against a poorly established evidence base. To resolve this tension and pave a path forward, a responsive approach requires a greater understanding of the current complexities associated with decision making under conditions of uncertainty. Within health systems that are adaptable to innovation, characterizing the presence and implications of decisional complexity is critical.

The implications of uncertainty on clinical and resource allocation decision making have been explored previously in context to precision medicine. Tomlinson et al. (2016) found that healthcare professionals report challenges communicating uncertainty, ensuring patient understanding, and managing patient expectations when obtaining consent for genomic sequencing [18]. These findings are echoed by other qualitative investigations eliciting provider experiences communicating genomic information and sequencing results to patients and research participants [19,20]. Further work has investigated payer and other health system stakeholder preferences for genomic testing in light of uncertain evidence. A recent discrete choice experiment found that hypothetical public preferences for genomic testing were sensitive to the degree of uncertainty in expected treatment alteration and survival gains [21]. A similar choice experiment distributed to US healthcare payers found that respondents placed higher utility for sequencing interventions leading to treatment change, and valued greater medical expert agreement regarding genomics-informed treatment alteration [22]. These findings support a call to reduce evidentiary uncertainty to enhance decision making and facilitate appropriate reimbursement of precision medicine interventions.

Combined, existing investigations have begun to illustrate the impact of uncertainty on clinical and resource allocation decisions in the context of precision medicine. Across this literature, evidence suggests a call to reduce uncertainty and build evidence to facilitate clinical and reimbursement decisions [21,22,23,24,25,26]. What is currently missing is an understanding of the challenges related to integrating precision oncology across publicly funded healthcare systems as it relates to managing uncertainty [19].

The objective of this qualitative interview study was to characterize experiences of clinicians and decision makers engaging with precision oncology and to identify key concerns, perceived implementation barriers, and recommendations to improve decisional processes. This work was conducted within a large-scale multi-year research project to generate evidence supporting genomics informed management of relapsed Lymphoid cancers. Within clinical contexts where interventions such as disease subtyping assays are under development and validation, yet carry uncertain clinical utility, capturing decisional challenges related to evidentiary uncertainty is critical. Without a comprehensive understanding of the challenges faced by those tasked with reimbursement and implementation of precision oncology, health systems will fail to ensure an adaptive and responsive approach to innovation. The reporting of this qualitative investigation follows the Consolidated Criteria for Reporting Qualitative Research (COREQ) checklist [27].

## 2. Materials and Methods

We conducted a series of semi-structured qualitative interviews with decision makers and clinicians with experience with precision oncology across Canada. Eligible clinicians were employed in cancer institutions with experience engaging with precision oncology in a clinical or research capacity. Eligible decision makers had professional experience with reimbursement or institutional approval of precision oncology innovations. An initial list of eligible participants was generated through searching publicly available institutional information. We applied a purposive snowball sampling approach, targeting pan-Canadian representation [25]. The study coordinator invited potential participants via email. Invitations included a summary of the research along with informed consent documentation. All participants provided written informed consent prior to their interview. At the time of the interview, the interviewer (SP, JD, or SC) provided a brief summary of the objective of the interviews and the types of questions that would be asked, as well as her involvement and role in the larger research project. A single interview was conducted for each participant.

Interview topic guides were co-developed by the multi-disciplinary research team consisting of health services and qualitative researchers, health economists, and clinician scientists. Decision-maker interviews were conducted first, followed by clinician interviews. Reflecting this order, the decision-maker interview guide was developed and subsequently adapted for clinician participants. The decision-maker interview guide was informed by reviewing published interview and focus group studies and guides addressing expectations and preferences for genomics [28,29,30,31]. In alignment with our research objectives, we prioritized key discussion topics. Broad discussion topics are summarized in Table 1 and address the following: (1) participant experience; (2) approval processes and implementation; (3) decision making under conditions of uncertainty; and (4) future directions, expectations, and recommendations.

Interview guides were intended to prompt key topics for discussion, while allowing for natural discourse between the interviewer and participant. All interviews were audio recorded and professionally transcribed. In advance of initiating interviews, the guide was pilot tested among the research team for clarity and length. Interviews were intended to last between 45 and 60 min in duration. During interviews, interviewers took minimal notes, and detailed field notes were documented immediately following each discussion. Following post-interview discussions among the research team, topic guides were modified throughout the interview process [32]. Initial interview topic guides are provided in Appendix A.

### Qualitative Analysis

Three female PhD and master’s level trained researchers with experience coding qualitative interviews and focus groups analysed focus group transcripts. Analysis begun immediately following the return of the first transcript, conducted using thematic analysis, and guided by a constant comparative approach [33]. A single analyst first read each transcript alongside the audio recording to ensure accuracy. De-identified transcripts were then uploaded into Nvivo coding software (QSR International Pty Ltd., version 12, 2018). Analysis begun with each analyst (S.P., J.D. or S.C.) applying an in vivo coding approach, wherein direct, brief excerpts are extracted to highlight key phrases and topics [31]. Using an iterative process to generate an initial list of in vivo codes, analysts then met to discuss extracted text and initiate the process of grouping excerpts into major and minor coding categories. Through frequent norming sessions, analysts applied a constant comparative approach to defining, expanding, and collapsing potential codes to refine the coding framework [34]. After each meeting, the coding framework was amended. After agreement was reached that the codebook was being applied consistently and no further codes were required, a single analyst (S.P. or J.D.) coded all remaining transcript files. Analysis of decision-maker transcripts was conducted by S.P. and S.C., and analysis of the clinician interview transcripts was conducted by S.P. and J.D.

Through the coding process, the primary analyst (S.P.) maintained detailed analytic memos. Analytic memo writing formed the basis of the identification of major and minor analytic themes. Themes were defined inductively through a process of further grouping and collapsing codes and frequent discussions among the research team. Given the potential for a divergence in views, analysts identified key differences in perspectives for integration into the analysis. Member checking was not conducted [35].

## 3. Results

Participants were recruited between June 2019 and May 2020. Eighty-one invitational emails were distributed, and 32 interviews were completed (decision maker *n* = 14, clinician *n* = 18). In total, 4 interviews were conducted in person (at cancer centres and an academic conference), and the remaining 28 interviews were conducted via telephone. S.C. conducted 12 interviews, S.P. conducted 7 interviews, and J.D. conducted the remaining 13 interviews. In two interviews conducted by S.P., J.D. was present as an observer for training purposes. Fifty percent of transcripts were coded in duplicate, and a single analyst coded remaining interviews. To our knowledge, four participants were familiar with one interviewer. Table 2 provides a summary of participant characteristics.

The majority of participants were recruited from British Columbia- (34%) and Ontario-based (28%) institutions. Clinician specialties spanned hematology, medical oncology, radiation, and surgical oncology. Decision makers reported experience with both provincial/institutional and pan-Canadian institutions, many of whom reported holding multiple academic, research, and decision-making appointments.

### 3.1. Emergent Themes

Through the qualitative analysis process, five major analytic themes were identified across both participant groups, defined as the following: (1) tempered optimism, (2) precision oncology to facilitate clinical decision making, (3) a responsive approach to public demand, (4) presence and implications of evidentiary uncertainty, and (5) collaboration as a prerequisite to decision making. Within the major theme titled presence and implications of evidentiary uncertainty, we further identified two emergent subthemes, namely (2a) driving evidence generation through novel approaches, and (2b) sustainable reimbursement through enabling early-stage evaluation, access, and re-evaluation. A summary and description of each major and subtheme is described in Table 3. Results across both clinicians and decision makers are reported together, with key divergence in perspectives described throughout.

### 3.2. Tempered Optimism

Discussions related to expectations and perceived benefits of precision medicine were frequently framed around a tempered or guarded optimism alongside anticipated barriers to clinical implementation (Quotes 1 and 2). This theme encapsulates the overarching sentiment related to the integration of precision oncology across health systems from the perspective of both clinicians and decision makers. Recognizing challenges with identifying adequate sample sizes to generate reliable impact estimates, decision makers cited evidentiary uncertainty as a mitigating factor to realizing benefit in the near term. Decision makers and clinicians used terms such as, “difficult,” “exciting,” “daunting,” and “challenging” to describe their experiences with resource allocation or clinical decisions related to precision oncology. Expressed frustrations were centered around a motivation to put forth recommendations to maximize health and resource efficiency in the absence of clear and directive evidence. Comparatively, decision makers voiced greater frustration around the lack of evidence able to inform reimbursement and access decisions (Quotes 1, 17, and 18). Both clinicians and decision makers reported experiencing decisional complexity, some voicing concern over implementation efforts in the absence of evidence to support value for money (Quotes 2 and 3). Direct quotes supporting the thematic analysis are presented in Table 4.

### 3.3. Precision Oncology to Facilitate Clinical Decision Making

A prominent theme across interviews was a motivation to engage with precision oncology to guide and support clinical decision making with information that could be used to inform decisions related to treatment alteration, de-escalation, and cessation (Quotes 4–7). For example, in the metastatic setting, one clinician spoke about maintaining quality of life through treatment de-escalation as a primary driver for engaging with precision oncology (Quote 4). In this sense, the concept of benefit or clinical utility was discussed broadly, sometimes outside the scope of survival gains. Similarly, compared to decision makers, clinicians more frequently valued returning information that could provide patients with accurate prognoses in the absence of clinical actionability.

Decision makers more often cited measurable health impacts as a prerequisite to reimbursement, applying a narrower conceptualization of benefit (Quotes 7 and 19). While decision makers acknowledged the potential personal value of prognostic information, there was a general hesitancy to allocate resources toward interventions unlikely to alter clinical outcomes. When prioritizing outcome measurement, decision makers reported a preference for evidence favouring clinical utility beyond immediate outcomes, with one individual citing diagnostic yield as a “poor indicator” of impact (Quote 19).

### 3.4. A Responsive Approach to Public Support

The role and influence of patient and public demand was discussed frequently throughout both clinician and decision-maker interviews. Clinicians reflected on experiences responding to patient support for precision oncology in clinical encounters, describing the time and effort spent in clinical appointments mitigating unrealistic expectations (Quotes 8 and 9). Throughout discussions pertaining to the importance and integration of patient and public voices in health technology evaluation, decision makers cited the need to be accountable to patient and public pressures (Quote 10, 11 and 26). Decision makers highlighted the need for a collaborative approach to evaluation processes, engaging those who are ultimately impacted by policy decisions (Quote 11, 24, and 26).

Both clinicians and decision makers endeavored to ensure a responsive approach to patient and public support for precision oncology while prioritizing the development of evidence informing appropriate resource allocation and implementation. Consistent across both groups was a desire to respond to patient and public expectations in a meaningful and evidence-informed manner.

### 3.5. Presence and Implications of Evidentiary Uncertainty

Evidentiary and decision uncertainty was central to discussions. Decision makers were frustrated by a lack of evidence allowing for robust evaluation, as well as evidence failing to capture meaningful health outcomes (Quotes 18 and 19). Similarly, there existed a tension across perspectives in terms of concern about premature implementation versus a desire to offer promising technologies to patients on the expecta-tion of benefit (Quote 12).

Among clinicians and decision makers, there was a persistent openness to a flexible approach to considering evidence to support decision making (Quotes 12–14 and 20). To reduce evidentiary uncertainty and facilitate timely decision making, participants discussed an enhanced role for evidence generated beyond the scope of randomized controlled trials. Citing challenges recruiting sufficient sample sizes as well as the rapidly innovative nature of precision oncology, clinicians welcomed a shift to integrate evidence generated through adaptive trials, real-world evaluations, and quasi-experimental investigations into decision making (Quotes 12–14). One clinician discussed improving collection of routine real-world data to support evidence generation (Quote 15). Decision makers also acknowledged the barriers associated with traditional experimental designs, some voicing a call for evidence beyond randomized controlled trials to respond to a need for up-to-date data supporting health technology assessment and reimbursement decisions (Quote 20).

Beyond data and evidence generation, participants similarly supported a nimble approach to the evaluation of precision oncology. One clinician described a life-cycle technology assessment (LC-HTA) process by which technologies are re-evaluated as real-world data emerge (Quote 16). In discussing the potential for a more iterative approach to evaluation, some decision makers cited challenges considering re-evaluation or disinvestment for reimbursed innovations shown to underperform in real-world settings (Quote 21). Others voiced concerns around balancing timely implementation against the need for comprehensive evaluation (Quote 2, 12, and 17). At times, clinicians expressed a greater tolerance for immature evidence, particularly when referring to patients with poor prognoses or the application of CGP within the pediatric setting (Quote 17). Central to these discussions was a call for flexibility in the approach to evaluation that is responsive to a changing evidentiary landscape, while ensuring quality of evidence thorough rigorous methods.

### 3.6. Collaboration as a Prerequisite to Decision Making

Persistent throughout interviews was a call for greater collaboration to increase decisional transparency, ensure equitable access, and integrate multiple perspectives into clinical and reimbursement decisions (Quotes 22, 23, 26, and 28–30). Challenges related to understanding decision-making processes and generating recommendations based on CGP findings were present at both the institutional and provincial level. Decision makers and clinicians described examples of siloed decision making and expressed frustration where they experienced a lack of collaboration across disciplines. Decision makers valued having genetic experts and clinicians involved in health technology assessments to provide content knowledge and expertise (Quote 28). Correspondingly, clinicians called for active engagement in the decision-making process to provide clinical insights and to contribute to the identification trade-offs required when considering reimbursement and access (Quote 23). Despite an acknowledged tension between decision makers and clinicians (Quote 22), both independently offered a call to navigate emergent challenges related to the reimbursement and implementation of precision oncology in collaboration.

Throughout interviews, precision oncology was discussed as a promising informational mechanism that is currently limited in its ability to inform downstream benefit. As discussed, participants referred to precision oncology HTA as sitting outside of the traditional evaluation landscape. As a result, there was an unfamiliarity with how to navigate a context wherein downstream impacts are highly heterogenous and challenging to estimate. Decision makers hoped to increase transparency across jurisdictions to determine how reimbursement decisions are made, using which evidence, and under varying competing budgetary constraints (Quotes 25 and 26). Key was the desire to acknowledge challenges related to resource allocation where patient and system impacts are poorly established, and to improve cross-jurisdictional conversations to approach such challenges using harmonized processes. For example, decision makers hoped to see greater alignment and consideration related to infrastructure and budget required to fund both testing and downstream intervention capacity (Quote 27). Similarly, clinicians questioned and were frustrated by the availability of sequencing technologies in the absence of available and accessible therapeutics.

Within patient care teams, some clinicians discussed challenges related to the integration of precision oncology into clinical care as a function of variable skillsets. These clinicians voiced a need for more accessible, up-to-date education resources to increase knowledge in genomics across disciplines (Quote 30). In this sense, clinicians acknowledged the emerging challenges associated with ensuring patients are provided with current and accurate information to assist decision making, alongside a prerequisite requiring ongoing education for care team to understand and communicate information generated through CGP. The need for continued education in response to increased clinical integration of precision was considered key to enhanced effective communication and implementation.

## 4. Discussion

The pace of precision oncology innovation coupled with patient and public demand is requiring clinicians and decision makers to manage uncertainties related to anticipated impact in the absence of clear evidence. As shown here, emerging challenges will require adaptive skillsets, the integration of a broader spectrum of evidence to support timely decision making, and innovative approaches to evaluation. This qualitative investigation presents a novel contribution to the literature by bringing together siloed voices to identify key challenges to decision making under conditions of evidentiary uncertainty. Alongside this, we identify avenues for collaboration and process improvements, through the development of a reflexive approach to evidence generation, evaluation, and collaboration.

Through the completion of 32 semi-structured qualitative interviews, we found that both clinicians and decision makers report a cautious optimism related to expected impact of precision oncology. Consistent was the perception that health systems are most likely to realize benefit through a transparent and collaborative approach to evidence generation, evaluation, and communication. Underpinning much of our findings was a desire to acknowledge and respond to patient, public, and governmental support for precision oncology, while ensuring that decisions are subject to the best available data and evidence. Our results are consistent with existing qualitative work reporting a guarded optimism related to precision oncology alongside a need to manage patient and public support for precision medicine and decisional complexity [19].

Despite a cautiously optimistic attitude toward downstream patient and population-level benefit, clinicians and decision makers differed in terms of valued endpoints informing practice and reimbursement. In general, decision makers were conservative in prioritizing reimbursement for sequencing strategies able to alter treatment trajectories with direct impacts to patient outcomes. Conversely, clinicians tended to voice a broader conceptualization of benefit. More frequently, clinicians spoke about the value of offering prognostic information to patients, and information that could be used to advance research and guide decisions outside the scope of treatment selection. In this sense, both clinicians and decision makers viewed precision oncology as a tool to support clinical decision making, yet clinicians placed greater value on the generation of information, regardless of its ability to directly alter patient outcomes. This finding aligns with recent work citing health system payer preferences for interventions able to incur benefit through treatment alteration [22].

Consistent with the emergence of investigations using real-world data and non-traditional trial designs to evaluate precision oncology, our findings offer a call to broaden the evaluative scope of evidence considered for reimbursement, particularly in clinical scenarios where randomized controlled trials are infeasible or uncommon [10,11,24]. Previously, Clausen et al. [25] found that Canadian health system stakeholders support the use of real-world evidence (RWE, e.g., administratively collected data) in cancer drug funding decisions, recognizing the limitations of and absence of high-quality randomized controlled trial (RCT) evidence. Authors further recommend a cultural shift away from RCTs as the sole gold standard for evidence informing novel oncology drugs. While support for leveraging real-world data (RWD) to generate RWE is emerging, challenges to realizing this goal persist. Within Canada, the siloed nature of provincial and territorial data holdings presents a barrier to the cross-jurisdictional identification of patients with similar genomic characteristics, as well as the sharing and linkage of individual patient-level data to support comparative effectiveness analyses. Current legislation requires institution- and project-specific data requests and approvals alongside substantial lags in data linkage and acquisition. Limitations on data sharing and linkage curtail the ability to conduct timely analyses using current and complete datasets able to generate effect estimates to support real-time decision making. While ours and other work appeals to the use of RWD to build out the evidence base for precision oncology, jurisdiction-specific barriers related to data infrastructure and data sharing currently hinder this effort.

Alongside an adaptive approach to evidence generation, our results highlight stakeholders’ desire for nimble and collaborative approaches to evaluating technologies throughout their life cycle. Defined as “a multidisciplinary process that uses explicit methods to determine the value of a health technology at different points in its life cycle,” [36] health technology assessment characterizes a systematic and transparent process alongside continuous evidence generation. As illustrated by this work, stakeholders expressed support for the integration of ongoing data and evidence generation to inform both clinical and reimbursement decisions. In practice, some participants noted challenges revisiting funding decisions, and that there lacks a clear and formal pathway by which underperforming technologies are re-evaluated in response to real-world evidence generated post-reimbursement. Efforts to respond to aspects of this unmet need are ongoing. In Canada, the Canadian Real-World Evidence for Value of Cancer Drugs (CanREValue) collaboration seeks to use real-world evidence in a consistent and standardized manner to support the re-evaluation of reimbursed oncology drugs [23]. In addition, the newly launched Canadian Network for Learning Healthcare Systems and Cost-Effective ‘Omics Innovation is working to build life-cycle evidence for effectiveness and cost-effectiveness, develop data infrastructure, and generate policy recommendations to support large-scale precision oncology initiatives across Canada [37]. Combined, these pan-Canadian initiatives are endeavouring to address data and system-level barriers to the generation of decision-grade evidence for precision oncology.

To realize the potential for RWE integration into reimbursement and disinvestment decisions for pre- and post-reimbursed drugs and technologies, preliminary efforts to enable re-evaluation processes are needed. Clinical and economic evaluations of precision oncology innovations able to inform access and reimbursement decisions begin with comprehensive data collection. To address data deficiencies within individual institutions, key stakeholders such as clinicians, information technology experts, data scientists, and evaluators are encouraged to collaborate to discuss institutional structures precluding the collection and use of RWD. Further, the integration of biostatisticians and data scientists into research teams conducting evaluations will enable rigorous and robust analyses of real-world data requiring, for example, quasi-experimental methods [38]. A multi-disciplinary approach to overcoming data and evidentiary deficiencies serves as a necessary prerequisite to generating decision-grade evidence. Broadening efforts to expand infrastructure to facilitate iterative evaluation processes will respond to support for the development of learning healthcare systems that are responsive to innovation [23,24,25,26].

Finally, our findings support a need for enhanced resources to assist clinical decision making [39,40]. Consistent with previous work, some clinicians reported challenges communicating information about genomic testing in light of inaccurate or unrealistic patient expectations. Clinician participants spoke about the time dedicated to increasing accurate knowledge, mitigating expectations, and communicating uncertainty to their patients. This finding aligns with previous investigations describing healthcare provider experiences and challenges communicating with patients about precision medicine [18,19,20]. Our results further highlight a call for enhanced decision supports and educational resources for patients tasked with highly complex and uncertain decisions. While decision support tools have been developed and validated for select clinical scenarios, such as the return of secondary findings [39] and management of hereditary cancer syndromes [41], our work supports further development across precision oncology contexts. Broadening the availability of educational resources has the potential to assist with managing patient expectations through increased knowledge, as well as to reduce decisional conflict and healthcare provider burden [42]. Combined, the sustainable integration of novel innovations that responds to health system stakeholder need requires both decision-grade evidence to ensure appropriate reimbursement and resources available to support patients and providers tasked with navigating complex discussions [15,24,43]. For this reason, future efforts directed toward the co-development and evaluation of decision support tools that consider patient- and clinician-valued outcomes that may include knowledge, anxiety, decisional conflict, consultation time, and satisfaction are warranted.

In summary, this work is intended to generate critical discussion around enabling nimble approaches to reimbursement, access, and uptake decisions for precision oncology. Across jurisdictions where decision makers are responsible for ensuring the appropriate direction of public resources in light of competing healthcare technologies, our results support a need to engage with those tasked with decision making under conditions of uncertainty. While challenges and recommendations reported here sit within the Canadian context, our findings may serve as a catalyst to engage in discussions around process improvements, decisional transparency, and enhanced infrastructure to support real-world evidence generation cross jurisdictionally. A critical next step towards addressing the breadth of decisional challenges within precision oncology involves expanding this work across privately and publicly funded health systems. A cross-jurisdictional understanding of barriers to the appropriate implementation of precision oncology will guide efforts to enhance infrastructure development and policy. Harmonization of data collection, evidence generation, and decision-making processes will further ensure equitable patient access to technologies demonstrating patient benefit alongside value for money.

### Limitations

This work should be interpreted considering its limitations. Firstly, the research team faced recruitment challenges achieving pan-Canadian representation. As a result, most of our interview participants were recruited from BC and Ontario. Additional recruitment efforts to gain insights representing greater geographic diversity may have yielded findings not captured by the current investigation. Despite this, recruitment continued until two qualitative researchers reached consensus that thematic saturation was reached. Secondly, although our intention was to capture pan-Canadian perspectives, all interviews were conducted in English and do not account for non-English language speaking stakeholders, potentially impacting the transferability of research findings. Thirdly, we did not report participant gender and therefore are unable to comment on corresponding variation in perspectives. Finally, although not a direct limitation, recruitment to thematic saturation was challenging. In total, 39% (32/81) of participants approached for participation completed an interview. Clinician recruitment occurred during the initial months of the COVID-19 pandemic, which presented recruitment delays and extended the duration of data collection. Future investigations seeking to elicit clinical feedback should consider potential recruitment challenges when planning research timelines and anticipated effort required to reach thematic saturation.

## 5. Conclusions

Within publicly funded health systems, the inappropriate direction of scarce healthcare resources has the potential for missed opportunities to improve patient health and well-being. As a result, a there exists a responsibility to ensure the acquisition and integration of the best available data and evidence to inform equitable reimbursement and clinical decision making. As evidenced by this work, welcoming innovation into a learning healthcare system will require system adaptations to ensure decisions are continually responsive to emerging evidence. Doing so will foster an environment wherein finite resources are directed toward innovations that yield acceptable patient benefit in light of required resources, while allowing for research and practice to exist simultaneously. In the absence of a mature evidence base informed by robust comparative effectiveness evaluations, challenges reported by our participants are likely to persist. What is now needed is a unified strategy centred on collaboration and a willingness to explore non-traditional evidentiary avenues using rigorous data collection and evaluation methods.

## Figures and Tables

**Table 1:** Key interview discussion topics.

Domain	Topic
Participant experience	Role and experiences with genetic testing (clinical, research, or decision making). Opinions about the clinical implementation of genomic testing, including predictive versus prognostic assays.
Approval processes and implementation	Conditions under which genomic tests are recommended (for reimbursement or patient access). Description of the process that genomic tests undergo in advance of approval for clinical use (decision makers only). Perceptions regarding how the current process works for evaluating novel oncology technologies and therapeutics (decision makers only).
Decision making under conditions of uncertainty	Experience managing evidentiary uncertainty. Evidentiary needs to support decision making. Concerns, challenges, and barriers to access and implementation. Data, evidence, and research or evidence gaps to be address.
Future directions, expectations, and recommendations	Perceived added value of CGP to patient car and expected benefits. Recommendations for reducing evidentiary uncertainty. Recommendations for health technology assessment (HTA) process improvement.

**Table 2:** Interview participant characteristics.

		N	%
Provincial representation (*n* = 32)	British Columbia (BC)	11	34%
	Ontario (ON)	9	28%
	Quebec	4	12%
	Alberta	3	9%
	Manitoba	1	3%
	Nova Scotia	1	3%
	Pan-Canadian (decision makers)	3	9%
Clinician Specialty (*n* = 18)	Adult hematology	9	50%
	Pediatric hematology-oncology	4	22%
	Medical oncology	3	17%
	Radiation oncology	1	6%
	Surgical oncology	1	6%
Decision-making experience (*n* = 14) *	Provincial/institutional	11	79%
	Pan-Canadian	5	36%

* Categories are not mutually exclusive.

**Table 3:** Themes identified through thematic analysis of transcripts.

Major Theme	Theme Description
Tempered optimism	Expected and anticipated value of precision medicine technologies to improve population-level and individual patient health.
Precision oncology to facilitate clinical decision making	The need for evidence to inform reimbursement decisions that prioritize access to innovations demonstrating improved survival and quality of life. Preference for the return of information to support patients navigating complex and uncertain decisions.
A responsive approach to public demand	Desire to respond to patient and public support for precision oncology in the clinic and at the reimbursement phase.
Presence and implications of evidentiary uncertainty	Experienced challenges related to the limited evidence able to provide robust estimates for clinical effectiveness and value for money, hampering clinical and reimbursement decision making Driving evidence generation through novel approaches; willingness to embrace evidence outside the scope of randomized trials to enable real-world estimates of clinical and economic value. Sustainable reimbursement through enabling early-stage evaluation, access, and re-evaluation; support for the adaptation of HTA frameworks to allow for re-evaluation of innovative technologies throughout their life cycle and integrate emerging evidence into updated decisions.
Collaboration as a prerequisite to decision making	A desire to work cross-disciplinarily to support a transparent and systematic approach to precision oncology reimbursement.

**Table 4:** Illustrative quotes.

Emergent Theme	N	Participant Quote	Participant ID
Tempered optimism	1	“I think it’s all very sexy and exciting. And we’re putting a lot of money in it and we’re also incentivizing industry to drive the drug development and research for smaller and smaller subsets of patient populations because we’re paying premium prices for the outputs of that, but perhaps at the expense of investment in other strategies, healthcare strategies that would provide much better value.”	(Decision maker (DM)-26)
	2	“… patients, they hear about the latest technology, and they’d like to have it, but, you know, when we jump ahead and just start using the technology and hope that someone else will evaluate it, it becomes harder …”	(Clinician (C)-3)
	3	“… there’s like a lot of parts of medicine that we’ve been doing for a long time that’s fairly standard across the provinces but this, because it’s new and complicated and it involves kind of setting up new systems, you know, it’s not really well standardized across the country.”	(C-7)
Precision oncology as to facilitate clinical decision making	4	“If someone’s functional, they’re interacting with their family, they’re doing the things that they enjoy, they’re part of our society, and that to me is more important. About that family aspect and society, it’s hard to quantify that, but … they’re an integral part of a family. And if they have quality, they maintain that.”	(C-1)
	5	“… some of the testing we ask for, for lymphoma needs to be done within the three weeks between two cycles of treatment … if it’s going to take weeks or months to get testing, then clinically its absolutely useless.”	(C-16)
	6	“Let’s say they’re waffling about whether they really want chemo at all. And then you can say to them, “Well, if you have the chemo, you’re probably still going to do poorly,” then that is actually something that helps them make their decision.”	(C-10)
	7	“The real question is whether this test is actually going to change somebody’s outcome …”	(DM-25)
A responsive approach to public support	8	“I think the perception of patients is sequencing is almost like a therapy and they don’t realize it’s a test … so how I phrase [program name removed] or any sequencing is we’re looking at the roadmap of your tumor to understand it better.”	(C-1)
	9	“I don’t know if the public understands that right now like when say patients with advanced cancer get a panel test, they’ll—only a very small number of them will have a mutation that could be targeted. And then amongst those, a very small number in practice get on to have some kind of genome-targeted treatment … I’m not sure how aware they are of those details. I think also that the research community and them, even the medical community is very in part, very enthusiastic about these techniques too. Which can maybe create false expectations for some.”	(C-2)
	10	“… when enough people start paying for something out-of-pocket, that’s a big red flag. So, the Province goes, “Should we be paying for this?””	(DM-24)
	11	“… HTA people want the information faster all the time. And we’re finding that these genetic technologies have actually slowed us down in the sense that they are complex, they take time to consider… we always do a patient preference, we do patient engagement with our HTAs, but with genetic HTAs there’s, you know, an added I think need to look at, you know, qualitative evidence on patient values and preferences. Also, to consider clinician’s perspectives, because a lot of the genetic tests will change management of the patient.”	(DM-23)
Presence and implications of evidentiary uncertainty	12	“It’s tough because I think we all want to have new technology that we think would be beneficial to our patients. And it’s costly and time consuming to run a randomized trial. And technology changes quickly so that in some cases if you did the trial, the technology that’s proven has now changed to some degree.”	(C-3)
	13	“So, I do think that new models of trials and data need to be put forward, and that’s all just part of the complexity that we have to tackle with precision oncology, so we can fight it.”	(C-2)
	14	“… diagnoses at a molecular level are proving really troublesome for HTA organizations to grapple with because it’s not your standard clinic trial paradigms anymore … I mean, so it’s fascinating, right, the need for adaptive clinical trials designed for basket trials or umbrella trails, and all these kinds of novel trial designs because of these new genomic tests. I mean it’s really challenging HTA organizations to sort of reimage what uncertainty means in clinical trials and in hierarchies of medical evidence. And challenging those very hierarchies, right?”	(C-5)
	15	“… let’s look at the individual level, do what’s best for the patient in front of us, but collect the data in a systematic way so that as we start doing this across the world you can actually pull the data and see where do certain patterns emerge of who responds, who doesn’t.”	(C-9)
	16	“… this concept of health technology management or life-cycle health technology assessment comes into play in that you do need to evaluate at various time points, depending on, you know, the patient populations that are exposed to the technology, potential incremental changes to the technology over time.”	(C-3)
	17	“Yeah, and well it’s a lot of these patients, like we’re talking mostly in the advanced setting, but a lot of these patients have a limited life expectancy, and they don’t have the luxury of time to wait for these drugs to come along.”	(C-4)
	18	“So, we’ve had topics submitted to us that once we get the scoping on them, we find that they are really in the experimental stage still, the research phase, and it is likely not a good—it is not optimal right now to do a health technology assessment because there is not a lot of evidence on it.”	(DM-23)
	19	“That’s an issue—challenge, we know most of these technologies are diagnostics and worst-case scenario, the measure of effectiveness is only something like a diagnostic yield. That’s terrible, because … It’s a poor indicator I think of clinical utility.”	(DM-24)
	20	“I think we just as HTA producers need to be a little bit more flexible on that, so that we can address the needs of the healthcare system without restricting ourselves or limiting ourselves to a certain type of information that is required.”	(DM-25)
	21	“But the problem is that when you’ve been offering a test for some five, six years, the decision we should stop paying for that, you’re in trouble because it’s much more difficult to withdraw something than to not implement it from the start.”	(DM-22)
Collaboration as a prerequisite to decision making	22	“So probably I think what needs to happen more is some more crosstalk between the policy makers and the clinicians. And I know there’s efforts, but there’s also mistrust. We’re like, “Oh, you guys are just a bunch of bean counters,” and they’re like, “You guys are sunny-eyed clinicians,” right?”	(C-1)
	23	“I mean, would we want access to all drugs? Sure. But realizing that someone has to make a decision, at least I can help inform that decision a bit better, like what are we potentially trading off by not funding this drug.”	(C-4)
	24	“I am completely sold on having lay people around the table. I think it’s essential, actually, because I think as academics, we don’t always realize how sterile we are in terms of the way we think about issues and evidence. And we need to be reined in sometimes and brought back to the real world … And it’s important; it’s absolutely necessary.”	(DM-24)
	25	“… it is very important that you have a good process that people will trust even if you don’t ultimately agree with the decisions, but you can trust that the decisions are made based on the best information, the best experts reviewing that information and a clear understanding of how decisions are made.”	(DM-26)
	26	“It’s a difficult space to be in. We need to promote, to I think open and transparent patient-informed, clinician-informed decision making just at a provincial if not a national level. We just need to promote that this is the expectation for decision making. That there’s clear processes, clear pathways that everybody can follow.”	(DM-21)
	27	“… even if we can find the budget to pay for the drug, can we even—are we prepared and do we have the infrastructure in place to actually even find, identify these eligible patients? Because do we have the money for the testing?”	(DM-20)
	28	“… critical success factor is having engaged and knowledgeable clinical experts who understand and are sympathetic of the notions of scarcity and just sensible decision making across the board … It can really help you understand the clinical pathways in that clinical area. Because inevitably, like, committees are populated by folks who don’t have that detailed knowledge of a single clinical area, typically.”	(DM-19)
	29	“So CADTH (Canadian Association for Drugs and Technologies in Health) is the only national organization, but the other ones are pretty tailored to their own provinces. So is there something we can do to work together and then just contextualize the information that would be specific for each province but kind of use much information that would be similar as possible.”	(DM-25)
	30	“So, it’s a challenge, it’s a very big challenge. Because there’s only—in a team of hematology/oncology, there’s probably just one or two members, maybe in a big team a few more, that have knowledge of the jargon that we use in genetic testing.”	(C-13)

## Data Availability

Due to the identifiability of interview participants, data will not be shared.

## Appendix A. Decision-Maker and Clinician Semi-Structured Interview Guides

**Table A1:** Decision-maker interview guide.

Domain	Interview Question *	Optional Prompt
Introduction and experience with genomic testing	Can you tell me about the work that you do in your organization?	What is your involvement in the review and approvals process for genomic tests in your organization? Are you involved in other aspects of the approvals process for what genomic tests are approved?
Decision-making processes	Can you briefly describe the process that genomic tests have to go through in order to get approved for use “in the clinic”?	Who is involved in development of the submission? What types of information are included in the proposals? Does the approval process look different for genomic versus usual care testing technologies? Are the standards for evidence different when you are considering genomic testing technologies? Does your organization have a framework to help decide whether to approve or reject proposals regarding the use of genomic tests?
	Can you discuss your experience reviewing proposals for prognostic-based genomic tests (as opposed to diagnostic or therapeutic tests)?	If yes, what sorts of criteria are considered when there is no corresponding change in treatment or impact on survival How does future clinical benefit factor into decision making—if at all? Is there a process to re-review the genomic test in the future when data are more mature or if new treatments do become available?
	Can you discuss whether the process submitting and reviewing proposals works well. If so, in what ways is it successful?	
	Can you describe any processes or frameworks that are used to review proposals for genomic testing in cancer?	Can you describe some ways in which you feel the process or framework works well?
Informational requirements and evidentiary uncertainty	What information would you need to make an informed decision about whether to invest in genomic testing for cancers?	What kinds of health or other outcomes would you need to see, to decide about approving a health technology like genetic testing?
	How, if at all, is uncertainty related to real-world impacts of a genomic test expressed in the proposals you review for new genomic tests?	What requirements or thresholds, if any, do you require before a genomic test will be approved?
Concerns and perceived barriers	What are the biggest challenges or barriers to the review, approval, and implementation process regarding genomic tests for cancer?	
Expectations and recommendations	What advice would you suggest for policy makers regarding the review and implementation process for prognostic tests? What would need to change to make the implementation process work better?	

* Wording of questions and optional prompts presented here are illustrative, in that interviewers used their own phrasing during each interview. The guides were used as a framework for discussion, rather than a script. Interviewers endeavoured to address each interview topic while allowing for natural discourse between the interviewer and participant.

**Table A2:** Clinician interview guide.

Domain	Interview Question *	Optional Prompt
Introduction and experience with genomic testing	Tell me about your experience with genomic testing in your practice.	What is the patient population that you care for? How often do you order genomic/genetic testing? How often do your patients approach you about genomic testing?
Initial opinions	What are your opinions about the clinical implementation of biomarker assays for cancer care?	
	The focus of emerging (lymphoid cancer) biomarker assays has tended toward providing patients with a more accurate prognosis and treatment de-escalation (e.g., stem cell transplants), rather than targeted treatments. What are your opinions about the clinical implementation of these kinds of tests?	What about tests that provide patients exclusively with information about their risk of disease relapse, or estimated survival?
Concerns and perceived barriers	Can you describe any concerns about the clinical implementation of these kinds of tests?	
	Can you tell me about any barriers that you foresee to the clinical implementation of these types of tests?	e.g., process barriers and barriers from patients’ perspectives
Benefits and expectations	In your opinion, what is the value that prognostic-based assays could add to the care of your patients?	
	Can you please describe the benefits that would you expect to see through the clinical implementation of prognostic-based assays?	What benefits would you need to see if you were going to refer your own patients for testing?
Implementation into current practice	Under which conditions, if any, would you foresee yourself referring your patients for prognostic-based assays?	What would need to change for you to support for the clinical implementation of prognostic-based assays for your patients?
	How would you (or how do you) go about making a decision about whether or not to recommend your patient for a genomic test?	What evidence do you look for? To what extent do you engage patients in the decision-making process? How do you weigh different forms of evidence?
Recommendations and future research directions	Are there specific research or evidence gaps that you would like to see addressed?	
	What areas of evidentiary uncertainty that you would like to see addressed before these sorts of tests can be implemented into the clinic?	

* Wording of questions and optional prompts presented here are illustrative, in that interviewers used their own phrasing during each interview. The guides were used as a framework for discussion, rather than a script. Interviewers endeavoured to address each interview topic while allowing for natural discourse between the interviewer and participant.
